# Effect of Three Commercial Formulations Containing Effective Microorganisms (EM) on Diflufenican and Flurochloridone Degradation in Soil

**DOI:** 10.3390/molecules27144541

**Published:** 2022-07-16

**Authors:** Paulina Książek-Trela, Ewelina Bielak, Dominika Węzka, Ewa Szpyrka

**Affiliations:** Department of Biotechnology, Institute of Biology and Biotechnology, University of Rzeszow, 1 Pigonia St., 35-310 Rzeszow, Poland; eb107477@stud.ur.edu.pl (E.B.); dw107523@stud.ur.edu.pl (D.W.)

**Keywords:** pesticides, diflufenican, flurochloridone, effective microorganisms, biodegradation

## Abstract

The aim of this study was to determine the influence of effective microorganisms (EM) present in biological formulations improving soil quality on degradation of two herbicides, diflufenican and flurochloridone. Three commercially available formulations containing EM were used: a formulation containing *Bifidobacterium*, *Lactobacillus*, *Lactococcus*, *Streptococcus*, *Bacillus,* and *Rhodopseudomonas* bacteria and the yeast *Saccharomyces cerevisiae*; a formulation containing *Streptomyces*, *Pseudomonas*, *Bacillus*, *Rhodococcus*, *Cellulomonas*, *Arthrobacter*, *Paenibacillusa*, and *Pseudonocardia* bacteria; and a formulation containing eight strains of *Bacillus* bacteria, *B. megaterium*, *B. amyloliquefaciens*, *B. pumilus*, *B. licheniformis*, *B. coagulans*, *B. laterosporus*, *B. mucilaginosus*, and *B. polymyxa*. It was demonstrated that those formulations influenced degradation of herbicides. All studied formulations containing EM reduced the diflufenican degradation level, from 35.5% to 38%, due to an increased acidity of the soil environment and increased durability of that substance at lower pH levels. In the case of flurochloridone, all studied EM formulations increased degradation of that active substance by 19.3% to 31.2% at the most. For control samples, equations describing kinetics of diflufenican and flurochloridone elimination were plotted, and a time of the half-life of these substances in laboratory conditions was calculated, amounting to 25.7 for diflufenican and 22.4 for flurochloridone.

## 1. Introduction

Effective microorganisms (EM) are a composition of interacting microorganisms of natural origin which are safe for the environment. They are used for production of natural and organic fertilizers and agents for revitalizing and nourishing the soil. EM improve the soil structure and fertility [1], as well as biodiversity, by enhancing the activity of soil microorganisms. They facilitate uptake of nutrients, thus influencing plant growth and development. They can increase crop yields and improve crop quality as well as accelerate the breakdown of organic matter from crop residues [2] and accelerate the decomposition of organic waste and pesticide residues and the composting process [3,4].

EM presence in the soil also contributes to a decrease in need for the use of chemical plant protection agents. This can be observed in cases where pesticides are replaced with microorganisms in the process of biological plant protection against pesticides in natural and organic agriculture [5]. Pests and pathogens are eliminated or controlled with natural strategies using beneficial microorganisms, which, e.g., initiate natural mechanisms of plant immunity, act antagonistically, or compete for space and nutrients [6,7].

The main EM are the bacteria *Bacillus* spp., *Streptococcus* spp., *Lactobacillus* spp., *Lactococcus* spp. (*Lactobacteria*), *Rhodopseudomonas* spp., and *Rhodobacter* spp. (photosynthesizing species), yeasts such as *Saccharomyces* spp. and *Candida* spp., Actinobacteria (*Streptomyces* spp.), and molds (*Aspergillus* spp.) [8].

According to many authors, the microorganisms may significantly contribute to the degradation of pesticides’ active substances [9,10,11]. Microorganisms are essential in bioremediation of pesticides, a cost-effective method for removal of pollutants from the environment. In biodegradation processes, pesticides are transformed into non-toxic metabolites used by microorganisms in metabolic processes. Enzymes catalyzing biochemical reactions, such as hydrolases, peroxidases, and oxygenases, play a key role in biotransformation mechanisms [12,13]. The degradation process efficiency depends on weather conditions, including soil temperature, pH, humidity, and composition. Pesticide elimination through biodegradation positively influences the fertility of agricultural soils [14,15]. Recently, many scientists isolated and cultured various microorganism species from the environment to conduct studies on pesticide degradation. The main types of microorganisms include *Bacillus*, *Pseudomonas*, *Flavobacterium*, *Micrococcus*, *Acinetobacter*, *Aerobacter*, *Alcaligenes, Flavobacterium*, *Clostridium*, *Actinomycetes*, *Penicillium*, *Aspergillus*, *Fusarium*, and *Trichoderma* [11,16,17,18].

The studies regarding persistent contaminants in soil, their fate, and dissipation are very important for environmental and human health. These pollutants could reach surface and ground water and also food products and then constitute a threat for consumers. It is very important to obtain knowledge of new, safe methods for soil remediation and environment protection. 

Diflufenican (IUPAC name *N*-(2,4-difluorophenyl)-2-[3-(trifluoromethyl)phenoxy] pyridine-3-carboxamide) is an herbicide widely used to control grasses and broad-leaved weeds in the cultivation of field peas, lentils, lupins, and winter cereals. According to the World Health Organization (WHO), it belongs to class III—slightly hazardous pesticide, but it could be harmful to aquatic life with long lasting effects [19,20].

Flurochloridone (3-chloro-4-(chloromethyl)-1-[3-(trifluoromethyl)phenyl] pyrrolidin-2-one) is a pre-emergence herbicide used to control a range of weeds in umbelliferous crops, cereals, and potatoes. It is classified as a slightly hazardous pesticide (WHO), but it could be toxic to aquatic organisms. This xenobiotic is suspected of damaging fertility or the unborn child [19,20].

The aim of this study was to present the effects of commercially available formulations containing EM on diflufenican and flurochloridone degradation in soil. Dissipation parameters and kinetic equations with correlation coefficients and half-lives (t_1/2_) were also presented. Additionally, changes in pH, oxidoreduction potential, and dehydrogenase activity (DHA) in the soil were analyzed. Soil was also screened for possible metabolites generated by the decomposition of these herbicides.

## 2. Results

The effects of commercially available formulations containing EM on degradation of active substances of two studied herbicides in the soil were described. Dissipation parameters and kinetic equations with correlation coefficients and half-lives (t_1/2_) were also presented. Additionally, metabolites generated by the decomposition of herbicides and the influence of soil microorganisms on the studied pesticides were also analyzed. 

### 2.1. Diflufenican

**Figure 1:**
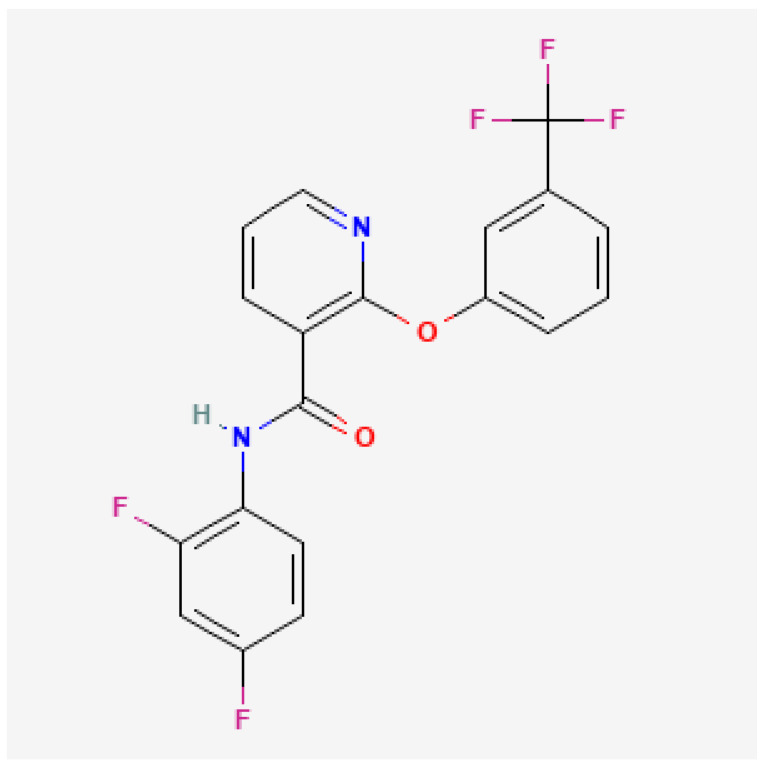
Chemical structure of diflufenican [19].

Diflufenican is a pyridine carboxamide herbicide belonging to Herbicide Resistance Action Committee (HRAC) Group 12 (carotenoid biosynthesis inhibitors) (Figure 1). It is a moderately persistent herbicide, non-mobile in soil.

Its possible metabolites found in the soil are 2-(3-trifluoromethylphenoxy) nicotinamide and 2-(3-trifluoromethylphenoxy) nicotinic acid. Another known metabolite is 2,4-difluoroaniline (Anaerobic) [20,21]. During a gas chromatography–mass spectrometry (GC-MS) full scan analysis of soil extracts (both non-derivatized and derivatized), none of these substances were found.

**Figure 2:**
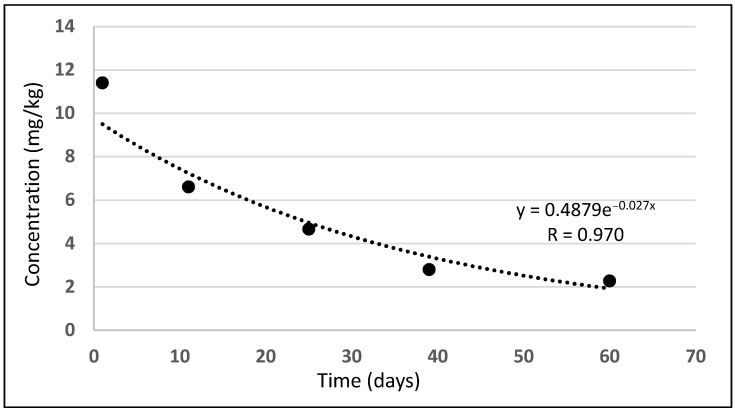
Diflufenican dissipation in the soil.

Figure 2 presents diflufenican’s dissipation in time. Diflufenican dissipated according to the equation: y = 0.4879e^−0.027x^ (R = 0.970). Its half-life was calculated, amounting to 25.7 days. 

Table 1 presents percentage degradation of diflufenican after 11, 25, 39, and 60 days from the application of EmFarma Plus™, Rewital PRO+, and BACILLUS VIP Probiotic Microorganisms versus the control.

Figures S1–S3 (Supplementary Material) present values of diflufenican levels in individual soil samples and its dissipation following application of formulations containing EM versus control samples. The control samples were soil samples with pesticide but without addition of EM.

### 2.2. Flurochloridone

**Figure 3:**
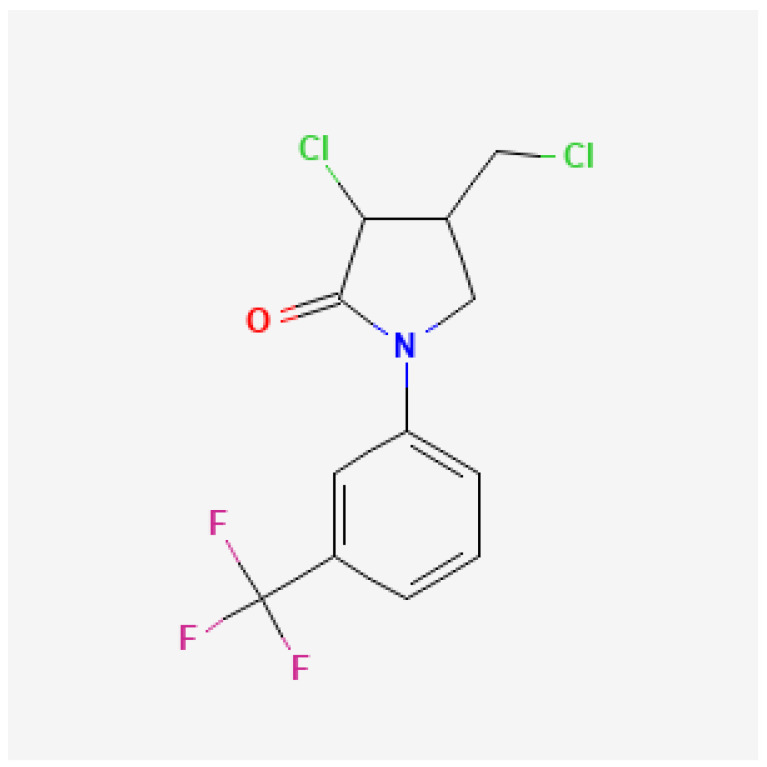
Chemical structure of flurochloridone [19].

Flurochloridone belongs to pyrrolidine 12 group (carotenoid biosynthesis inhibitors) according to the HRAC (Figure 3). It is a moderately persistent herbicide. There is no information about its mobility in soil. 

Its possible soil metabolites are 4(chloromethyl)-3-hydroxy-1[3(trifluoromethyl)phenyl] pyrrolidin-2-one and 4-(chloromethyl)-1-[3(trifluoromethyl)phenyl] pyrrolidin-2-one. Another known metabolite is (4*R*,*S*)-4(chloromethyl)-1-[3-(trifluoromethyl)phenyl]pyrrolidin-2-one [20,21]. During a GC-MS full scan analysis of soil extracts (both non-derivatized and derivatized), none of these substances were found.

**Figure 4:**
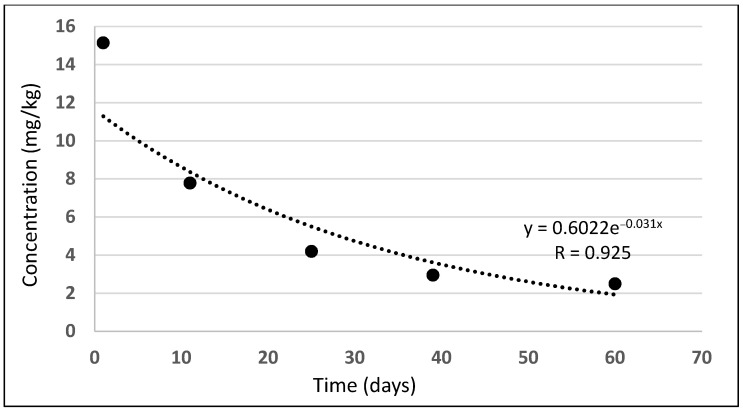
Flurochloridone dissipation in the soil.

Figure 4 presents flurochloridone dissipation in time on Days 1, 11, 25, 39, and 60 of the study. Flurochloridone dissipated according to the equation: y = 0.6022e^−0.031x^ (R = 0.925), achieving its half-life of t_1/2_ = 22.4 days.

Table 2 presents a percentage degradation of flurochloridone after 11, 25, 39, and 60 days from the application of EmFarma Plus™, Rewital PRO+, and BACILLUS VIP Probiotic Microorganisms versus the control.

Figures S4–S6 (Supplementary Material) present values of flurochloridone levels in individual soil samples and its dissipation following application of formulations containing EM versus control samples.

### 2.3. GC-MS Full Scan Analysis

After derivatization of soil extracts, TMS derivatives of palmitic acid were found in analyzed samples and in the blank sample. In all analyzed soil samples, TMS derivatives of palmitoleic acid, oleic acid, glyceryl monopalmitate, and glyceryl monostearate were found and were not detected in the blank soil sample. 

### 2.4. Soil Parameters: pH, Oxidoreduction Potential, and DHA

On the first day, the pH of all soil samples was 7.4 for diflufenican and 7.3 for flurochloridone. When EM formulations were added to samples with diflufenican, the pH of all studied samples decreased significantly, even by 1.2 pH units. In samples with flurochloridone, after EM formulations were added, pH was on the same level as in the control samples. The last measurement, on Day 60, is an exception, as a significant drop in pH was observed for all samples, caused by the long experiment and closing of samples in containers (3 weeks from the last measurement).

In all samples with diflufenican, the oxidoreduction potential was at a slightly higher level in the study samples versus the control ones, except for Day 60, and ranged from 266 (Day 25, control sample) to 358 (Day 60, sample containing Rewital PRO+). In samples with flurochloridone, the oxidoreduction potential was higher when compared to the control samples on Day 11; on Days 25 and 39, it remained at a similar level as that in the control samples, and ranged from 266 (Day 25, sample with EmFarma Plus™) to 346 (Day 60, sample with Rewital PRO+).

The water content was measured in the samples on Day 1 and amounted to 67%. Then, on successive days, the water content was 70–74% for control and study samples.

**Figure 5:**
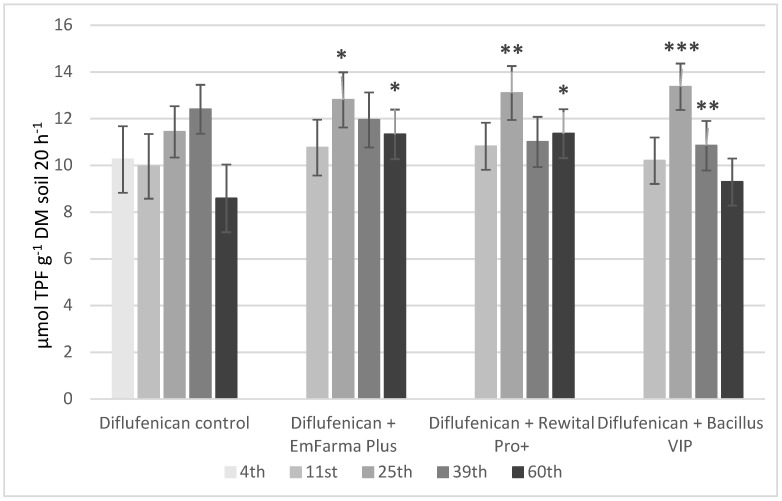
Changes in the DHA of the soil with diflufenican and following application of formulations with EM in time. Statistically significant *p* values are shown as *p* < 0.05 (*), *p* < 0.01 (**) and *p* < 0.001 (***).

**Figure 6:**
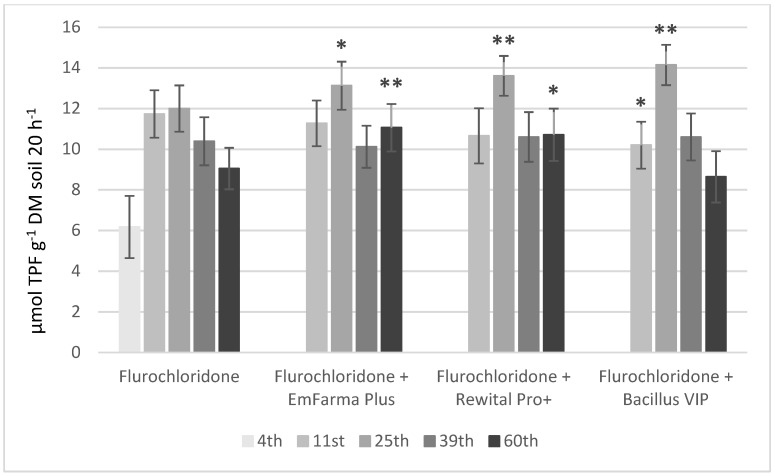
Changes in the DHA of the soil with flurochloridone and following application of formulations with EM in time. Statistically significant *p* values are shown as *p* < 0.05 (*) and *p* < 0.01 (**).

DHA activity changes are presented in Figure 5 and Figure 6. Application of diflufenican and flurochloridone resulted in increases in DHA activity in control samples, especially on the 25th and 39th days in the case of diflufenican and on the 11th and 25th days in the case of flurochloridone. On the 25th day, DHA activity of soil samples with herbicides and EM preparations was significantly higher than in control samples. On the 39th day, DHA activity in soil with diflufenican and EM preparations decreased compared to control samples. It confirms our results that diflufenican disappeared more slowly in soil with the addition of EM preparations, probably due to the change in soil pH. On the 39th day, DHA activity in soil with flurochloridone and EM preparations was on the same level as in the control samples. On the 60th day, DHA activity decreased in samples with herbicides. The enzyme activity in samples with herbicides and EmFarma Plus™ and Rewital PRO+ preparations was significantly higher than in the control samples.

Our results confirm that the herbicide or its metabolites may stimulate the activity of microorganisms in the first period, while the activity of DHA decreases with dissipation of the active substance [22].

## 3. Discussion

### 3.1. Kinetics of Active Substances Diflufenican and Flurochloridone and Their Disspation and Half-Lives in Soil 

#### 3.1.1. Diflufenican

The experiments were conducted using formulation Legato 500 SC, containing diflufenican as an active substance in the quantity of 500 g of active substance/L.

One day after application, diflufenican residues amounted to 11.401 mg/kg and dissipated in accordance with an exponential equation: y = 0.4879e^−0.027x^ (R = 0.970), achieving a half-life of t_1/2_ = 25.7 days. On the last day of the study, diflufenican residues amounted to 2.267 mg/kg.

The database of pesticide properties specifies that half-lives of diflufenican in the soil are 41.4–318 days in laboratory conditions and 224–621 days in the field [20].

Rouchaud et al. [23] calculated the half-life of diflufenican in the soil in the field, and it amounted to 101 days, while Rouchaud et al. [24] specified the half-life of the herbicide in the soil in a pear orchard, and it amounted to about 65 days for the soil treated with diflufenican for the first time and every year for 3 years.

The diflufenican decomposition rates in the soil differed in pH, coming from Kirton (soil of pH 7.8) and from Wellesbourne (soil of pH 6.8). DT25 of the pesticide was determined, and for two soils it amounted to 29.5 weeks. Diflufenican degraded very slowly, without a change in the degradation rate over time [25].

Conte et al. [26] found no acceleration in diflufenican metabolism with the increase in the pesticide level. The herbicide was used in two fields, before sowing, every year for 4 years. Soil samples were analyzed directly after application every year and at 6 and 12 months after application. Each year, wheat and maize were cultivated in the fields as the main crop and the succeeding crop, respectively. The analysis did not show any residues in any of the wheat or maize plant parts in quantities exceeding the limit of quantification (0.001 mg/kg). No herbicide accumulation year after year was demonstrated. The DT50 value was calculated, amounting to ca. 14 days. 

In our experiments, diflufenican dissipated faster (lower t_1/2_ value) than reported by other researchers, and this may have resulted from the experimental conditions differing from the field ones, i.e., the temperature maintained at a level of 22 ± 1 °C, and especially, a high moisture content of 67–74%.

#### 3.1.2. Flurochloridone 

The experiments were conducted using formulation Racer 250 EC, containing flurochloridone as an active substance in the quantity of 2500 g of active substance/L. 

One day after the formulation application, flurochloridone residues amounted to 15.141 mg/kg and dissipated according to the exponential equation: y = 0.6022e^−0.031x^ (R = 0.925), achieving its half-life of t_1/2_ = 22.4 days. On the last day, the 60th day of the study, flurochloridone residues amounted to 2.487 mg/kg. The flurochloridone dissipation parameters achieved in our experiments are correlated with data obtained by other researchers.

The database of pesticides properties specifies that half-lives of diflufenican in the soil are 22.8–180 days in laboratory conditions and 11–65 days in the field [20].

Rouchaud et al. [27] calculated its half-life in the soil in the field, and it amounted to 41 days. The next year, they repeated this study in the same field and the t_1/2_ increased to 50 days. 

In another experiment, flurochloridone dissipation in potatoes and soil on China’s Qinghai plateau (Haixi, Haibei and Haidong) was studied using the modified QuEChERS method combined with liquid chromatography—mass spectrometry (LC-MS/MS). The flurochloridone half-life was calculated, and it amounted to 25.7, 27.7, and 19.3 days in the soil and 8.1, 10.2, and 7.0 in potato leaves in three districts (Haibei, Haixi, and Haidong), respectively. The pesticide degraded at the fastest rate in the Haidong region, in which rainfalls were relatively heavy. Final flurochloridone residues amounted to <0.001, 0.005–0.013, <0.001 and 0.007–0.023 mg/kg in potato leaves, roots, tubers, and soil, respectively, and did not exceed the maximum acceptable level of residues (0.1 mg/kg) [28]. 

Sharipov et al. [29] assessed the half-life for flurochloridone in three soils from different agricultural regions of the Czech Republic where sunflower are cultivated. The soils differed in pH values, CaCO_3_ content, organic matter, and contents of clay, silt, and sand. The half-life, t_1/2_, amounted to 38 days for the soil of the Haplic Chernozem type (Suchdol), 46 days for the soil of the Haplic Fluvisol type (Dobromerice), and 51 days for the soil of the Arenic Regozem type (Volarna). The highest degradation of the herbicide occurred in the Arenic Regozem soil, characterized by the highest pH and the highest CaCO_3_ and sand content when compared to other studied types of soil. That soil was also characterized by the lowest organic matter and silt content.

### 3.2. Influence of EM in Formulations on Herbicide Degradation in the Soil in Laboratory Conditions 

Following a treatment with formulations containing EM, EmFarma Plus™, Rewital PRO+, and BACILLUS VIP Probiotic Microorganisms, the influence of those microorganisms on herbicide degradation in the soil was studied. The literature review concerning decomposition of pesticide residues indicates a great interest in the use of biological methods for degradation of active substances by bacteria, fungi, and yeasts. 

#### 3.2.1. Diflufenican

After application of diflufenican and formulation EmFarma Plus™, a degradation at a level of 2.4% to −36.5% was observed compared to the control samples. When Rewital PRO+ was applied, the degradation ranged from 1.4% to −35.5%, while after application of BACILLUS VIP Probiotic Microorganisms, the degradation ranged from 7.2% to −38%. A statistically significant difference was noted for EmFarma Plus™ and Rewital PRO+. No statistically significant differences were observed for BACILLUS VIP Probiotic Microorganisms (Table 1 and Figures S1–S3 Supplementary Material). The results of the conducted experiment indicate that EM had a significant effect on the diflufenican degradation in the soil, but they proved to be opposite to the expected ones.

To this date, there have been several reports on the influence of organic fertilizers applied to soil on diflufenican degradation and one paper on microorganism influence on the diflufenican decomposition.

The degradation was probably inhibited by a significant drop in the soil pH following application of the biological formulations (by 1 pH unit versus the control). According to Houot et al. [30], soil of pH below 6.5 is characterized by a slower metabolic degradation.

Similar results were obtained during a study performed in winter wheat in Belgium. To analyze diflufenican dissipation in field conditions, before sowing, the soil was treated with green fertilizer, cow manure, or pig slurry. The herbicide formulation was used at a dose of 250 g/ha. The diflufenican residues were tested 11 times during 281 days after the treatment. The diflufenican half-life was calculated, and it was 101, 116, 215, and 176 days for the control soil, and the soil treated with green fertilizer, pig slurry, and cow manure, respectively. Organic fertilizers increased durability of the herbicide and its metabolites 2(2-[3-trifluromethyl) phenoxy]-3-pyridinecarboxylic acid, 3(*N*-2,4-difluorophenyl)-2-hydroxy-3-pyridincarboxyamide, and 4,2-hydroxy-3-carboxypyridine. This study confirms the results of our research—increased persistent of diflufenican after application of biological preparations to the soil. After 6 months, the effect of the organic fertilizers was less pronounced and diflufenican and its metabolites in the soil were at similarly low levels [23].

Diflufenican dissipation in field conditions was analyzed in the soil on which winter wheat was sown. Herold^®^ formulation (Spain) was used, containing 20% diflufenican and 40% flufenacet. The field was divided into plots: control soil (S), soil treated with the herbicide (S + H), control soil with spent mushroom substrate treatment (S + SMS), soil treated with the herbicide and SMS (S + H + SMS), soil treated with green compost (S + GC), and soil treated with the herbicide and GC (S + H + GC). Pesticide residues were analyzed 0, 45, 145, 229, and 339 days after the herbicide application. At the beginning of the experiment, diflufenican levels in the control and in the treated soil ranged between 2.24 and 2.81 µg/g of dry matter. The diflufenican levels decreased in S and S + GC 45 days after the treatment; however, no significant reduction was noted in S + SMS, probably due to a different initial diflufenican absorption by the soil. After 339 days, diflufenican residues in the topsoil were >65% [31].

Other studies were conducted in two types of soil, clay loam and sandy loam. The soils were enriched with different types of organic material, using municipal solid waste (MSW) and cow manure (CM). In both cases, the diflufenican content decreased gradually. The study was conducted for 250 days. At the end of the experiment period, the diflufenican content decreased significantly, by 25.5% in clay loam and by 41.2% for the soil treated with CM and MSW, while in the sandy loam, the diflufenican content dropped by 32.5% and 50.2% for CM and MSW, respectively, versus the control group [32]. These results also confirm faster dissipation of diflufenican in soil with lower organic content. 

The influence of fungi, *Fusarium oxysporum* PP0030, *Paecilomyces variotii* PP0040, and *Trichoderma viride* PP0050 on dissipation of pesticides, including diflufenican, was also studied. These species proved to be valuable as active microorganisms decomposing pesticides and having a very high capability for biotransformation of target pesticides. The highest level of diflufenican degradation was achieved by *F. oxysporum*, followed by *P. variotii* and *T. viride*. After 120 days, the maximum degradation of diflufenican by *F. oxysporum* amounted to 74.7% [33].

#### 3.2.2. Flurochloridone

After application of flurochloridone and formulation EmFarma Plus™, degradation at a level of 4.4% to 19.3% was observed compared to the control samples. When Rewital PRO+ was applied, the degradation ranged from 7.1% to 30.5%, while after application of BACILLUS VIP Probiotic Microorganisms, the degradation ranged from 17.8% to 31.2%. A statistically significant difference was noted for Rewital VIP+ and BACILLUS VIP Probiotic Microorganisms. No statistically significant differences were observed for EmFarma Plus™ (Table 2 and Figures S4–S6 Supplementary Material).

To this day, only one report has been published on the influence of organic fertilizers on flurochloridone degradation.

Rouchaud et al. [27] studied the flurochloridone dissipation in field conditions in Melle, Belgium. The herbicide (Racer 25CS, Zeneca, Cambridge, UK) at a dose 500 g/ha on the soil was applied 6 months before planting potatoes. Then, the field was divided into plots, and each plot was treated with a different natural fertilizer: cow manure, pig slurry, or green manure (yellow mustard, Emergo type). Control plots were not treated with the organic fertilizers. All treatments (the plots treated with the fertilizers and the control plots) were performed in four fields each (four replicates). The studies were conducted for 2.5 months. The flurochloridone half-life was calculated and it amounted to 48, 67, and 74 for the soil treated with green manure, pig slurry, and cow manure, respectively. In the control plot, t_1/2_ amounted to 41 days. The experiment was repeated in the same field in the subsequent year. Each plot was treated with the same organic fertilizer. The herbicide half-life was verified again. The t_1/2_ increased and amounted to 50 days for the control, and 58, 80, and 70 days for the soil treated with green manure, pig slurry, and cow manure, respectively. In both studies, the highest increase in the herbicide degradation rate was achieved with the green manure. After the potatoes were harvested, the soil in all plots was examined, and flurochloridone was not found again (limit of quantification of 0.01 mg/kg). This results confirm our findings that EM significantly contribute to dissipation of flurochloridone in soil.

## 4. Materials and Methods

Three commercially available formulations containing EM were used in the study:

EmFarma Plus™ (ProBiotics, Bratuszyn, Poland)—a formulation revitalizing the soil. A natural biostimulator of plant growth and development. It effectively accelerates decomposition of organic matter and increases availability of nutrients, mainly nitrogen, to plants. Microorganisms contained in the formulation, including phototropic bacteria (more resistant to sun rays), interact with each other and replace pathogenic microflora. Its main component is stock cultures of live microorganisms, SCD ProBio Plus^®^. These are concentrated mixes of live microorganisms produced in a natural fermentation process with participation of beneficial microorganisms commonly found in the environment. The formulation includes bacteria: *Bifidobacterium*, *Lactobacillus*, *Lactococcus*, *Streptococcus*, *Bacillus*, and *Rhodopseudomonas* and yeast *Saccharomyces cerevisiae*, organic sugar cane molasses, revitalized non-chlorinated water, rock salt, and a mineral complex. Molasses is a medium for microorganisms and is rich in numerous components, including micronutrients, that may have an advantageous effect on plant growth [34].

Rewital PRO+ (BIOGEN, Warszawa, Poland)—a formulation revitalizing the soil—contains beneficial bacteria for restoring and maintaining microbiological balance in the soil degraded by, e.g., the use of chemical plant protection agents. The formulation includes bacteria: *Streptomyces*, *Pseudomonas*, *Bacillus*, *Rhodococcus*, *Cellulomonas*, *Arthrobacter*, *Paenibacillus*, and *Pseudonocardia* and the starter medium. These bacteria facilitate nutrient absorption by plants, improve the soil structure, and support mineralization of residues in the soil, reducing incidence of plant diseases. Microorganisms included in the formulation support and appropriately control processes of the organic matter decomposition in the soil, which significantly influence the nitrogen, phosphorus, and sulfur compound management in the soil [35].

BACILLUS VIP Probiotic Microorganisms (AGROBIOS, Nowy Tomyśl, Poland) is a formulation that improves soil fertility by stimulating plant immunity and growth. The formulation contains eight bacteria strains from the *Bacillus* genus which are highly resistant to the environmental stress caused, e.g., by high temperature, exposure to UV light, lack of water, and high salt content: *B. megaterium*, *B. amyloliquefaciens*, *B. pumilus*, *B. licheniformis*, *B. coagulans*, *B. laterosporus*, *B. mucilaginosus*, and *B. polymyxa*, as well as organic sugar cane molasses and revitalized water. Each of eight bacteria strains in the formulation provide numerous benefits. The most of them include increased bioavailability of nutrients: phosphorus, nitrogen, potassium, and iron, production of growth substances (amino acids, enzymes, phytohormones), stimulation of plant and root growth and plant germination, and increasing plant resistance to pests and diseases (production of antibiotics or fungicides) [36].

### 4.1. Reagents

The reagents used for analyses of herbicides and their metabolites in soil included: hexane and acetone of the analytical grade (Witko, Łódź, Poland), petroleum ether for GC (Chempur, Piekary Śląskie, Poland), analytical standards of herbicides (Sigma-Aldrich, St. Louis, MO, USA), sorbents for extraction by the QuEChERS method (magnesium sulfate, MgSO_4_; sodium chloride, NaCl; sodium citrate, Na_3_C_6_H_5_O_7_; disodium citrate sesquihydrate, C_12_H_18_Na_4_O_17_) (Chempur, Poland), and sorbents for clean-up by the dispersive solid phase extraction according to the QuEChERS method (primary and secondary amines PSA (Agilent, Santa Clara, CA, USA) and MgSO_4_ (Chempur, Poland), Silylating mixture II according to Horning (BSA + TMCS + TMSI 3:2:3) for the GC derivatization (Sigma-Aldrich, USA), and phosphate buffer (60 mM, pH = 7) (Chempur, Poland). The reagents used for DHA analysis included: methanol LC-MS (Honeywell Speciality Chemicals Seelze GmbH, Seelze, Germany), 2,3,5-Triphenyltetrazolium chloride (Sigma-Aldrich, USA), 1,3,5-Triphenyl tetrazolium formazan (Tokyo Chemical Industry, Tokyo, Japan). 

### 4.2. Soil Sample Preparation

The soil used in experiment was a universal one, recommended for horticultural crops. It consists of low moor and high moor peat, pine bark, sand, perlite, dolomite, and mineral fertilizers. Soil parameters: pH 6.0–7.3 and a salt level of 0.5–1.0 g KCl/dm^3^ (PPUH Zielona Oaza I, Brzozów, Poland).

The experiment was conducted for t2 months, from October to December, at a constant ambient temperature of 22 ± 1 °C and the constant soil humidity of 67–74% (Figures S7–S12, Supplementary Material).

400 g samples of soil were weighed into transparent propylene containers of 2 L each. 80 mL of the formulation Legato 500 SC (ADAMA, Warszawa, Poland) [37], containing diflufenican as an active substance, were added to each of twelve soil samples, and 80 mL of the formulation Racer 250 EC (ADAMA, Warszawa, Poland) [38], containing flurochloridone as an active substance, to each of twelve other soil samples. Legato 500 SC is used to control weeds in cereals before or soon after their emergence. It is absorbed through leaves and partly through plant roots. Racer 250 EC is used against mono- and dicotyledon annual weeds in winter cereals and in vegetables. The plant absorbs it through roots and cotyledons of germinating weeds.

Currently, diflufenican and flurochloridone are registered and approved for use as an active substance of herbicides in the European Union [39].

Legato 500 SC (active substance diflufenican 500 g/L) at a dose 0.1 mL/L and Racer 250 EC (flurochloridone 2500 g/L) at a dose 0.2 mL/L were applied in the form of spraying and mixing with the soil [37,38]. All samples were prepared and analyzed in three replicates (three containers per each sample). 

On the next day, 12 h after the treatment, soil samples were collected and analyzed for pesticide residues. Then, on Day 4 of the experiment, formulations containing effective microorganisms, EmFarma Plus™, Rewital PRO+, and BACILLUS VIP Probiotic Microorganisms were added to the samples in accordance with the study plan provided in Table 3. Water was added to control samples, containing only studied pesticides. Samples were mixed thoroughly. All samples were prepared and analyzed in three replicates (three containers per each sample). The blank samples were soil with water.

The samples for pesticide analyses were collected 1, 11, 25, 39, and 60 days after the herbicide application. Before sampling, each soil was mixed thoroughly with a laboratory spoon. In soil samples, water content was measured by a weighing method after drying at 105 °C (S-40, Alpina, Konin, Poland) [40]. Additionally, during sampling, pH, and the oxidoreduction potential were measured with a digital meter ORP/pH AD14 (ADWA, Szeged, Hungary). The pesticide concentration was calculated for the soil dry mass. All soil samples were stored in stable laboratory conditions.

Figures S7–S12 (Supplementary Material) present changes in pH, the oxidoreduction potential, and the moisture content for the soil samples during successive days of the experiment.

### 4.3. GC-MS Analysis of Pesticides Residues and Possible Metabolites

Herbicides in the soil were analyzed using the modified standardized method PN-EN 15662:2018-06 entitled “Foods of plant origin. Multimethod for the determination of pesticide residues using GC- and LC-based analysis following acetonitrile extraction/partitioning and clean-up by dispersive SPE. Modular QuEChERS-method” [41,42]. The modified method was developed and validated in our laboratory for food matrices and soil samples. Validation was carried out with the determination of such parameters as: linearity, recovery, precision, limits of quantification, the working range of the method, and its uncertainty [42].

In brief, 5 g of soil were weighed into a 50-mL polypropylene centrifuge tube, and 10 mL of water and 10 mL of acetone:hexane (1:4 *v*/*v*) mixture were added. The tube content was vortexed (BenchMixerTM, Benchmark, Sayreville, NJ, USA) for 1 min. Then, a mixture of QuEChERS extraction salts was added. The tube content was vortexed in a mixer for 1 min and then centrifuged at >4000 rpm (5804R, Eppendorf, Hamburg, Germany) for 5 min. 5 mL of the organic phase were transferred to a 15 mL polypropylene centrifuge tube that contained sorbents for cleanup by the dispersive solid phase extraction method.

A 7890A gas chromatograph (Agilent Technologies, Palo Alto, CA, USA) equipped with a mass detector, model 7000 (GC-MS/MS QqQ), was used to analyze herbicide residues in soil extracts and to determine possible metabolites in Quechers extracts and after its derivatization by the TMS solution.

Before the chromatographic analysis, triphenyl phosphate (TPP) was added to the samples as an internal standard. The samples were monitored in the dynamic multiple reaction monitoring (dMRM) mode. For diflufenican, quantitative determination was performed for fragmentation 266 → 238.1 *m*/*z* (collision cell energy 15 eV), and qualitative confirmation was performed for fragmentations 266 → 246 *m*/*z* (15 eV) and 394 → 266 *m*/*z* (10 eV). The following fragmentation reactions were monitored for flurochloridone: 311 → 173.9 *m*/*z* (collision cell energy 15 eV), 186.8 → 158.9 *m*/*z* (10 eV, quantitative ion) and 145.1 → 95.1 *m*/*z* (15 eV) [42]. The chromatograms of soil samples are shown in Figures S13 and S14 (Supplementary Material).

Additionally, possible metabolites of pesticides were determined in the soil samples. The analysis was performed for Quechers extracts and for extracts subjected to the derivatization process. The sample extract was evaporated under the nitrogen stream, and 100 µL of Silylating mixture II was added according to Horning (BSA + TMCS + TMSI 3:2:3) for GC derivatization (Sigma-Aldrich, USA). The sample was left at the room temperature for 30 min. Then, 500 µL of hexane was added and it was thoroughly mixed for 30 s on the vortex; 1 mL of phosphate buffer (60 mM, pH = 7) was added, and the sample was shaken manually for 1 min. The samples were left for 30 min until separation of the phases. The top layer (the hexane phase) was transferred into the autosampler vial and analyzed in the full scan mode in the range 40–450 *m*/*z*. The following analysis parameters were used: injection of samples in a splitless mode, injected volume—1 μL, carrier gas—helium (flow 1 mL/min), ionization mode—electron (−70 eV), and the temperatures of 250 °C for the injector and transfer line, 300 °C for the ion source, 150 °C for the quadrupoles, and 70–280 °C for the oven.

### 4.4. Assessment of DHA

The DHA activity was determined in the soil samples according to Casida et al. [43], Tabatabai [44] and Wołejko et al. [22]. Briefly, 6 g of soil was weighed into 50 mL propylene centrifuge tube, 4 mL of distilled water and 1 mL of 3% aqueous solution of 2,3,5-triphenyltetrazolium chloride (TTC) were added. Samples were incubated in the dark at 37 °C for 20 h. After incubation, 20 mL of methanol was added to the samples, vortexed for 1 min (BenchMixerTM, Benchmark, USA), centrifuged (5804R, Eppendorf, Hamburg, Germany), and filtered. The DHA activity was measured at 485 nm with spectrophotometer Cary 300 Bio (VARIAN, Palo Alto, CA, USA). All results were expressed in micromoles of 1,3,5-triphenyltetrazolium formazan (TPF) per gram of dry soil per 20 h.

### 4.5. Statistical Analysis of Results

The Student test (Excel Microsoft 365 program) was used to determine statistically significant differences between samples, with and without biological preparation added, for each sampling day. Statistically significant *p* values are shown in Figure 5, Figure 6 and Figures S1–S6 (Supplementary Material) as *p* < 0.05 (*), *p* < 0.01 (**) and *p* < 0.001 (***). 

## 5. Conclusions

Generally, EM are considered as biodegradation factors for different chemical pollutants. They are used to accelerate dissipation of organic contamination and to remediate and revitalize soil. In our study, we obtained divergent results for two different pesticides. It was demonstrated that commercial EM intended for soil revitalization significantly influenced the degradation of herbicides. For diflufenican, the obtained results were opposite to the expected ones. All studied formulations containing EM reduced the diflufenican degradation level, from 35.5% to 38%, due to an increased acidity of the soil environment and increased durability of that substance at lower pH levels. In the case of flurochloridone, all studied EM formulations increased the degradation of that active substance by 19.3% to 31.2% at the most. The results of our study confirm the need to continue and develop research on the influence of individual EM on the fate of xenobiotics in the environment.

## Figures and Tables

**Table 1:** Percentage diflufenican degradation following application of formulations containing EM versus control samples (the content of herbicide in control samples, on each sampling day, was assumed as 100%; ‘−’ means that the degradation rate decreased).

Number of Days after Formulation Application	Degradation (%)		
	EmFarma Plus™	Rewital PRO+	BACILLUS VIP Probiotic Microorganisms
11	−36.5 **	−35.5	−32.6
25	7.2	1.4	7.2
39	3.5	−25.0 *	−38.0
60	2.4	−9.1	−10.0

*p* < 0.05 (*) and *p* < 0.01 (**).

**Table 2:** Percentage degradation of flurochloridone following application of formulations containing EM versus control samples (the content of herbicide in control samples, on each sampling day, was assumed as 100%).

Number of Days after Formulation Application	Degradation (%)		
	EmFarma Plus™	Rewital PRO+	BACILLUS VIP Probiotic Microorganisms
11	18.3	7.1	22.0
25	4.9	27.7	29.8
39	4.4	17.9	17.8
60	19.3	30.5 *	31.2 *

*p* < 0.05 (*).

**Table 3:** Study plan.

Samples Number	Title 3
1–3	soil + diflufenican
4–6	soil + diflufenican + EmFarma Plus™
7–9	soil + diflufenican + Rewital PRO+
10–12	soil + diflufenican + BACILLUS VIP Probiotic Microorganisms
13–15	soil + flurochloridone
16–18	soil + flurochloridone + EmFarma Plus™
19–21	soil + flurochloridone + Rewital PRO+
22–24	soil + flurochloridone + BACILLUS VIP Probiotic Microorganisms

## Supplementary Materials

The following supporting information can be downloaded at: https://www.mdpi.com/article/10.3390/molecules27144541/s1, Figure S1: Diflufenican levels in individual soil samples and its dissipation following application of EmFarma Plus™ versus the control samples. Statistically significant *p* value is shown as *p* < 0.01 (**); Figure S2: Diflufenican levels in individual soil samples and its dissipation following application of Rewital PRO+ versus the control samples. Statistically significant *p* value is shown as *p* < 0.05 (*); Figure S3: Diflufenican levels in individual soil samples and its dissipation following application of BACILLUS VIP Probiotic Microorganisms versus the control samples. No statistically significant differences were observed; Figure S4: Flurochloridone levels in individual soil samples and its dissipation following application of EmFarma Plus™ versus the control samples. No statistically significant differences were observed; Figure S5: Flurochloridone levels in individual soil samples and its dissipation following application of Rewital PRO+ versus the control samples. Statistically significant *p* value is shown as *p* < 0.05 (*); Figure S6: Flurochloridone levels in individual soil samples and its dissipation following application of BACILLUS VIP Probiotic Microorganisms versus the control samples. Statistically significant *p* value is shown as *p* < 0.05 (*); Figure S7: Changes in the soil pH levels with diflufenican and following application of formulations containing effective microorganisms during successive days of the experiment; Figure S8: Changes in the soil pH levels with flurochloridone and following application of formulations containing effective microorganisms during successive days of the experiment; Figure S9: Changes in a moisture content of the soil with diflufenican and following application of formulations with EM in time; Figure S10: Changes in a moisture content of the soil with flurochloridone and following application of formulations with EM in time; Figure S11: Changes in the oxidoreduction potential of the soil with diflufenican and following application of formulations with EM in time; Figure S12: Changes in the oxidoreduction potential of the soil with flurochloridone and following application of formulations with EM in time; Figure S13: Chromatogram of soil sample with diflufenican (retention time for diflufenican—27.854 min, and internal standard—triphenyl phosphate, TPP—27.794 min); Figure S14: Chromatogram of soil sample with flurochloridone (retention time for flurochloridone—20.515 min, and internal standard—triphenyl phosphate, TPP—27.794 min).
