# Supplementary material for: Effect of Three Commercial Formulations Containing Effective Microorganisms (EM) on Diflufenican and Flurochloridone Degradation in Soil
Source: Molecules. 2022 Jul 16;27(14):4541. doi: 10.3390/molecules27144541 (PMC9319521; doi:10.3390/molecules27144541)
Supplement: Supplementary file 1 [file molecules-27-04541-s001.zip › molecules-1821487-supplementary.pdf]

Supplementary material

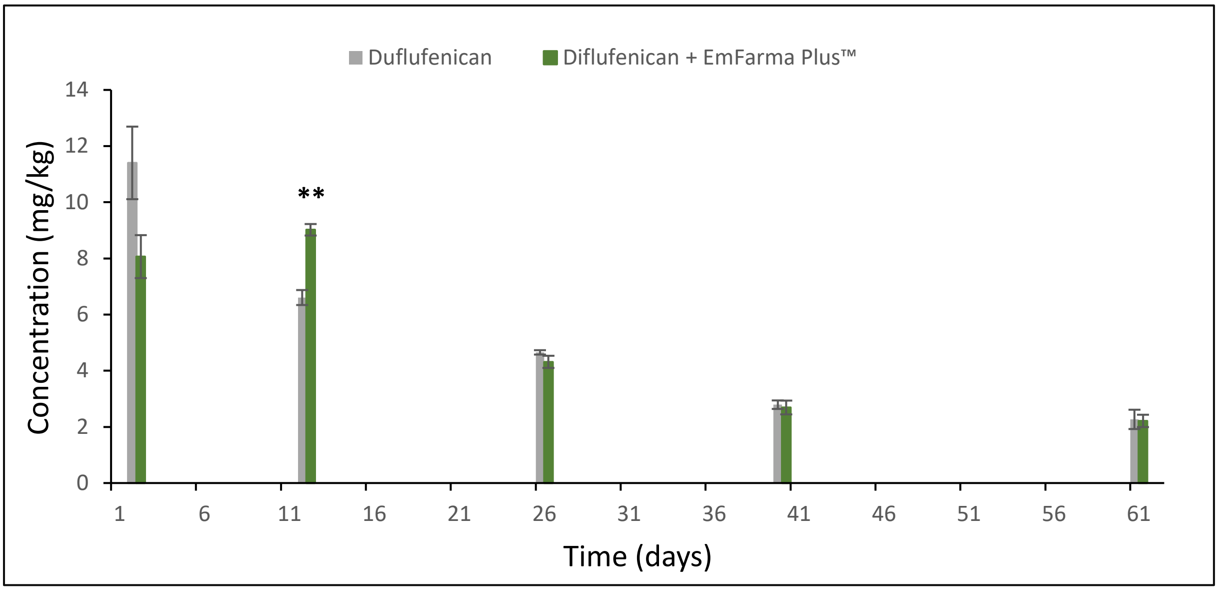

Figure S1: Diflufenican levels in individual soil samples and its dissipation following application of EmFarma Plus™ versus the control samples. Statistically significant p value is shown as $p < 0.01$ (**).

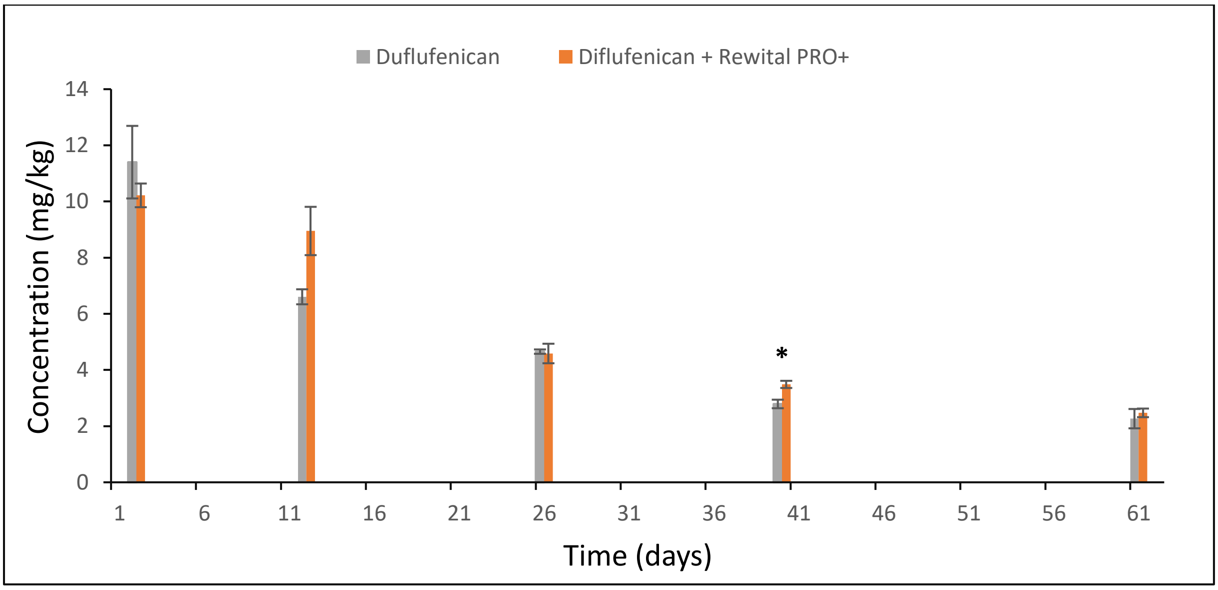

Figure S2: Diflufenican levels in individual soil samples and its dissipation following application of Rewital PRO+ versus the control samples. Statistically significant p value is shown as $p < 0.05$ (*).

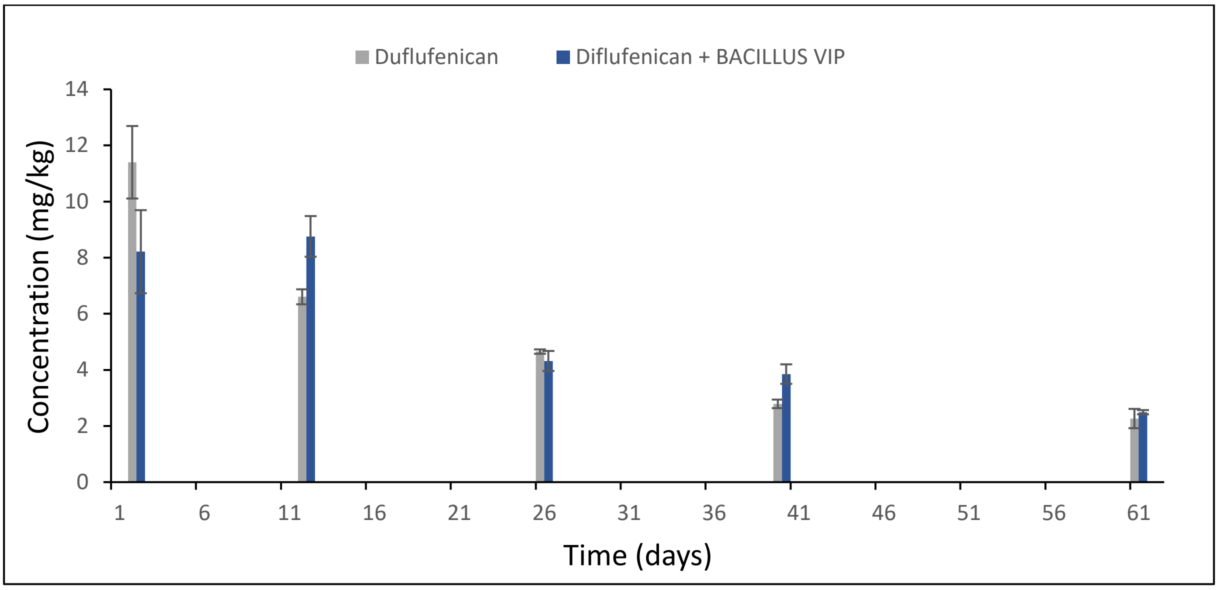

Figure S3: Diflufenican levels in individual soil samples and its dissipation following application of BACILLUS VIP Probiotic Microorganisms versus the control samples. No statistically significant differences were observed.

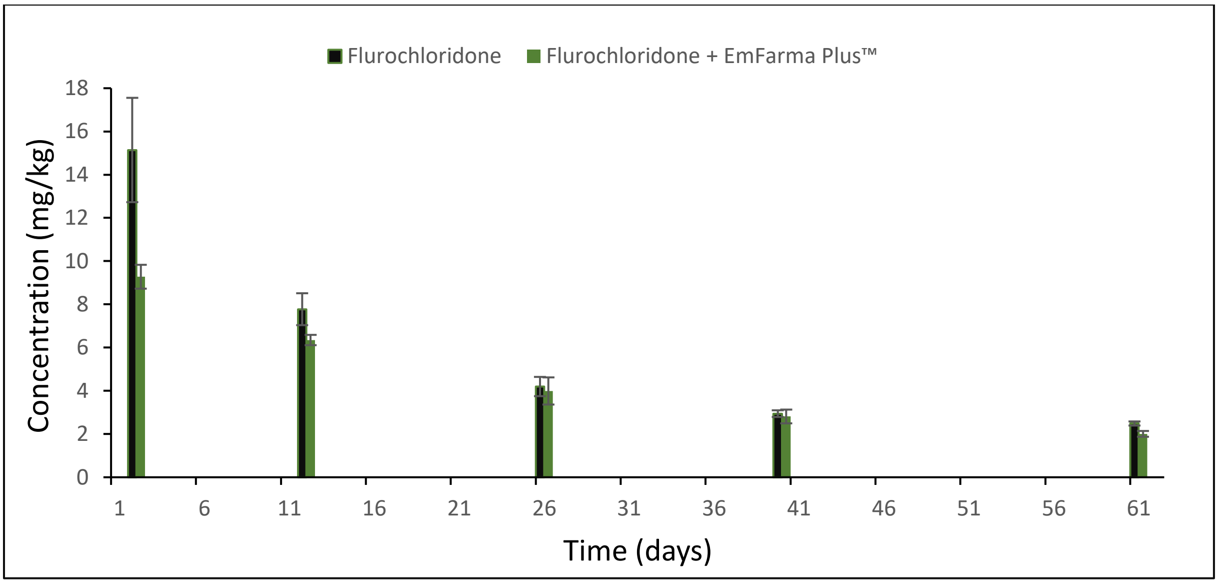

Figure S4: Flurochloridone levels in individual soil samples and its dissipation following application of EmFarma Plus™ versus the control samples. No statistically significant differences were observed.

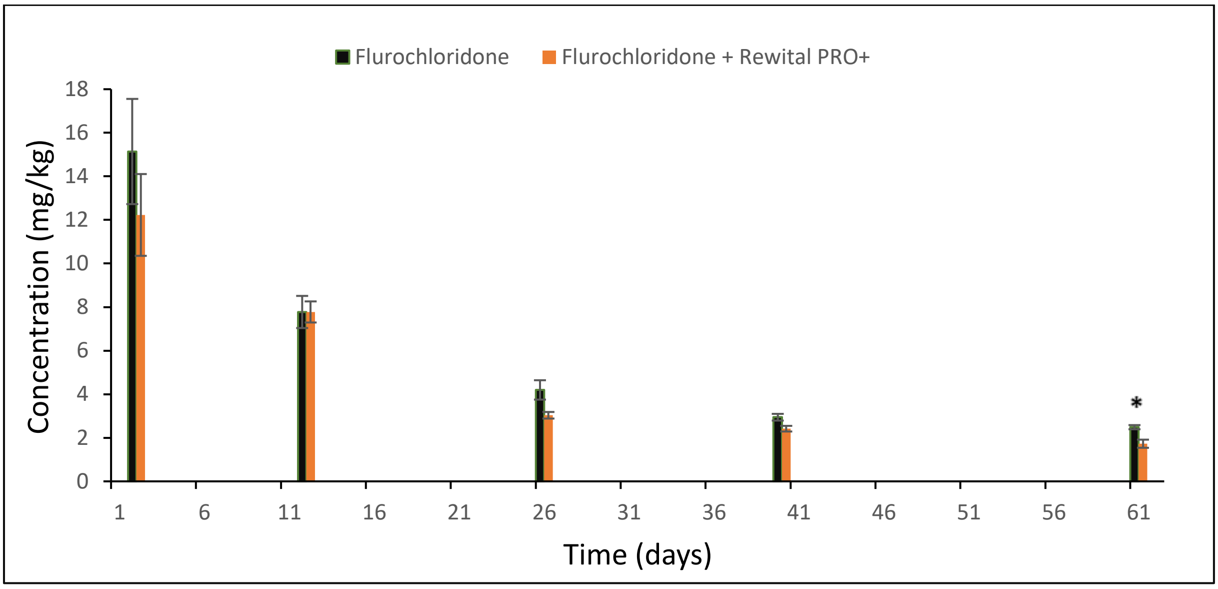

Figure S5: Flurochloridone levels in individual soil samples and its dissipation following application of Rewital PRO+ versus the control samples. Statistically significant p value is shown as $p < 0.05$ (*).

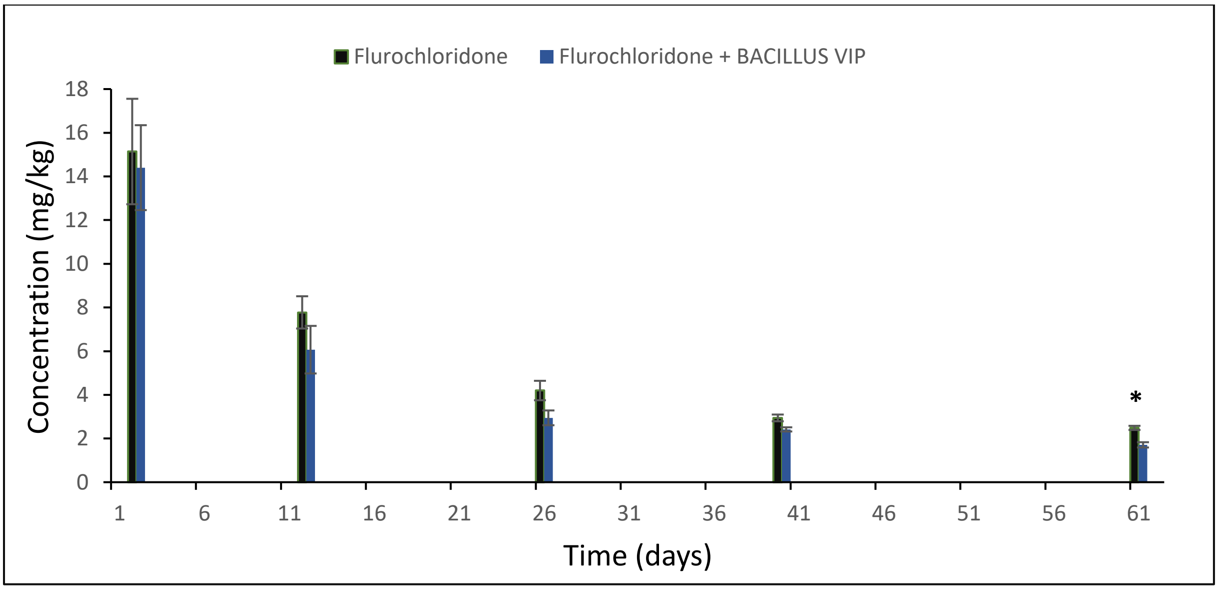

Figure S6: Flurochloridone levels in individual soil samples and its dissipation following application of BACILLUS VIP Probiotic Microorganisms versus the control samples. Statistically significant p value is shown as $p < 0.05$ (*).

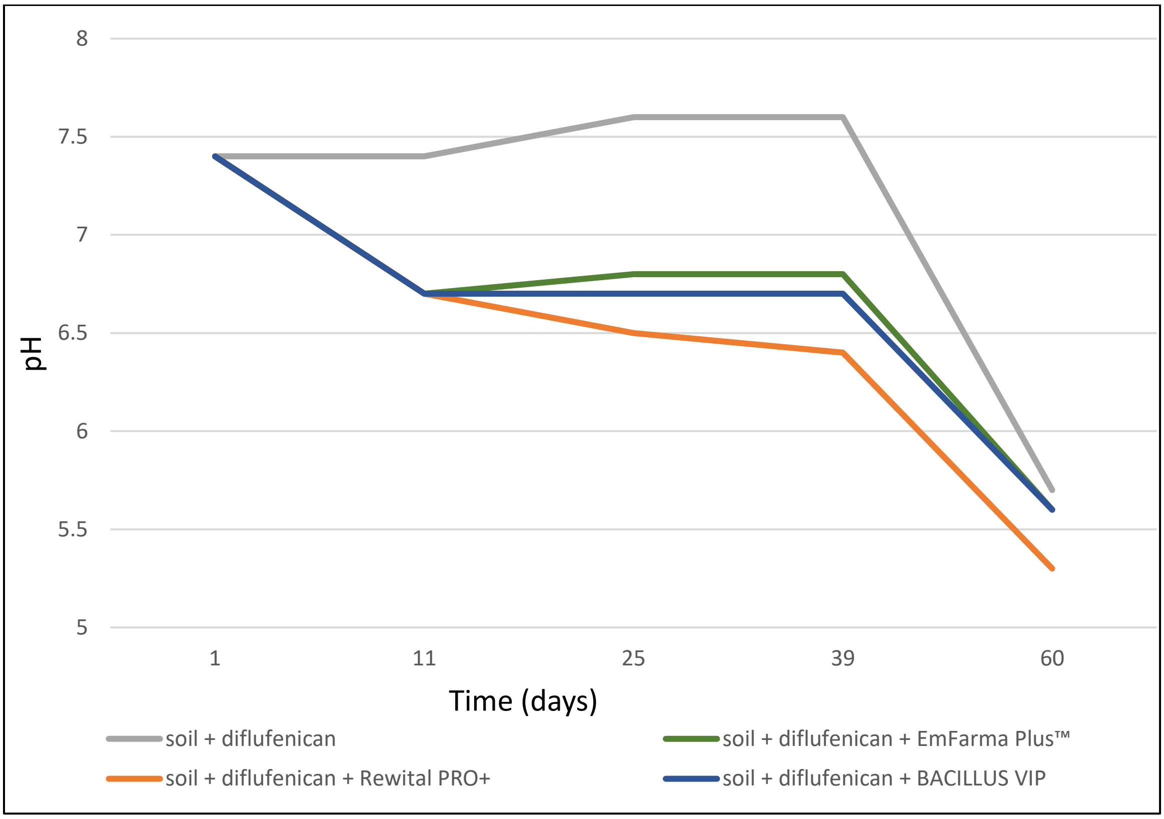

Figure S7: Changes in the soil pH levels with diflufenican and following application of formulations containing effective microorganisms during successive days of the experiment.

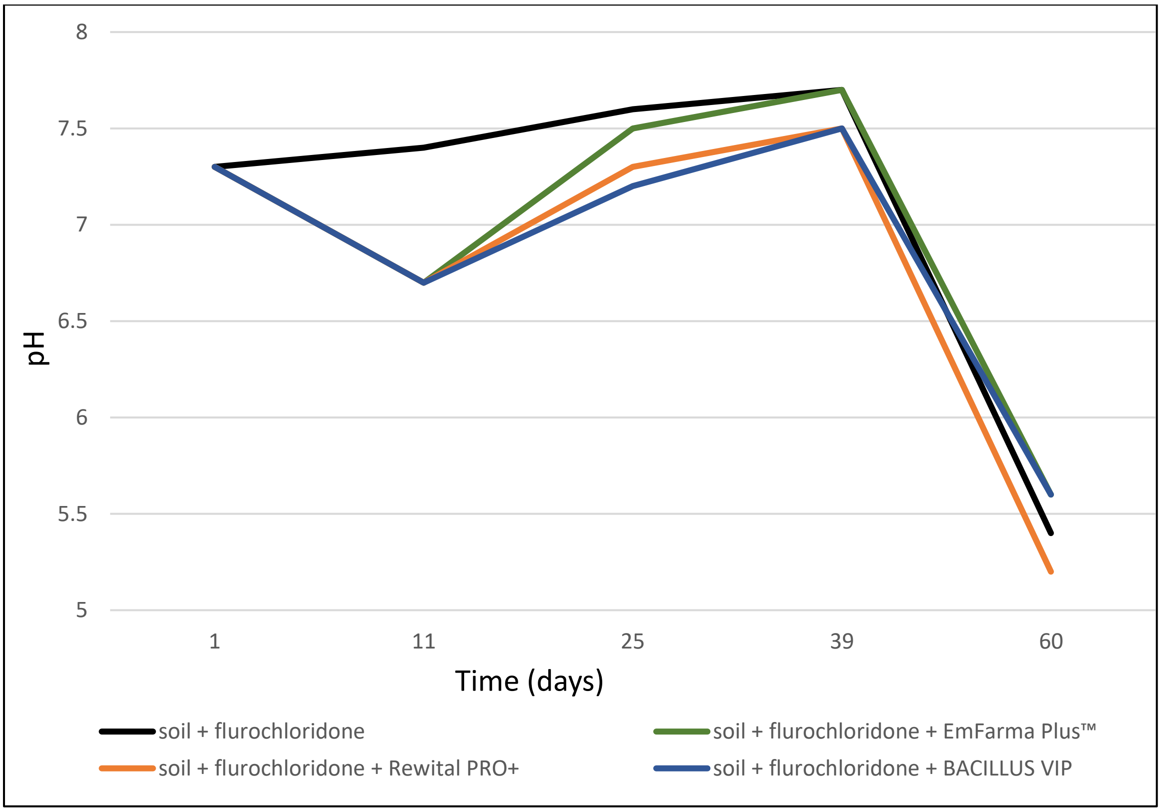

Figure S8: Changes in the soil pH levels with flurochloridone and following application of formulations containing effective microorganisms during successive days of the experiment.

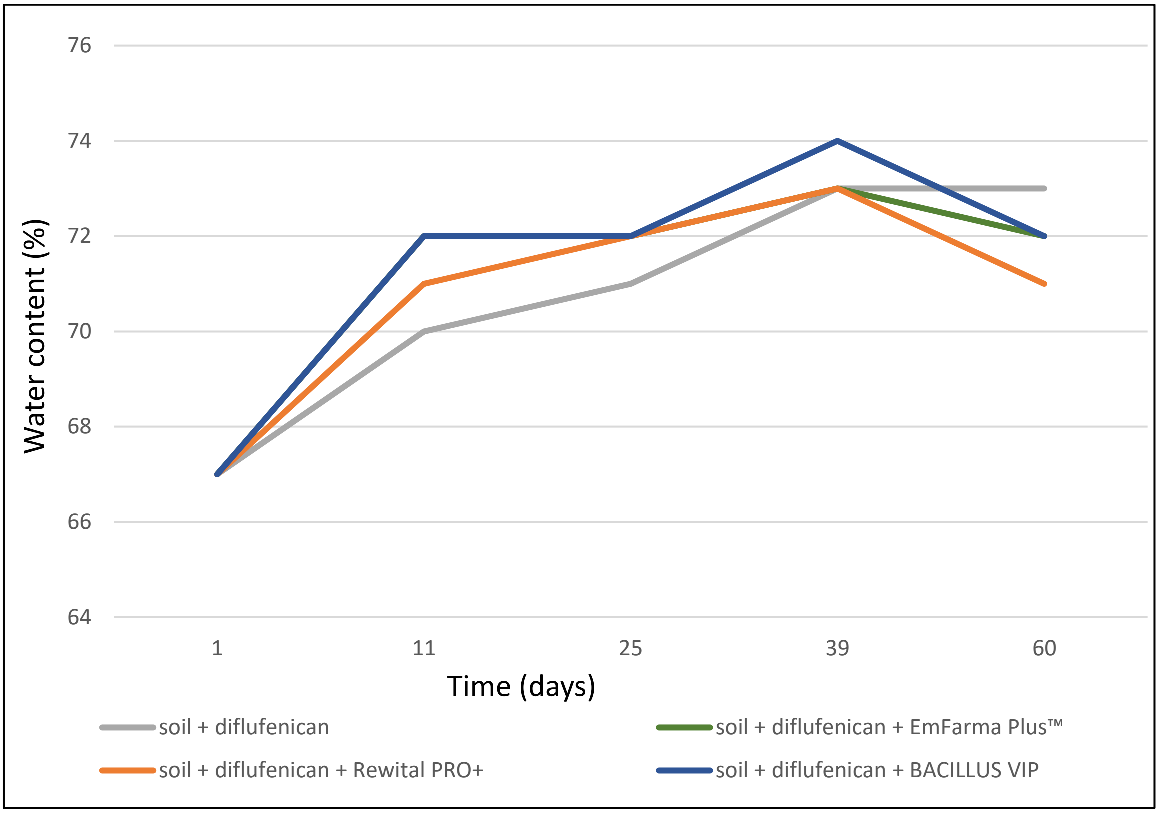

Figure S9: Changes in a moisture content of the soil with diflufenican and following application of formulations with EM in time.

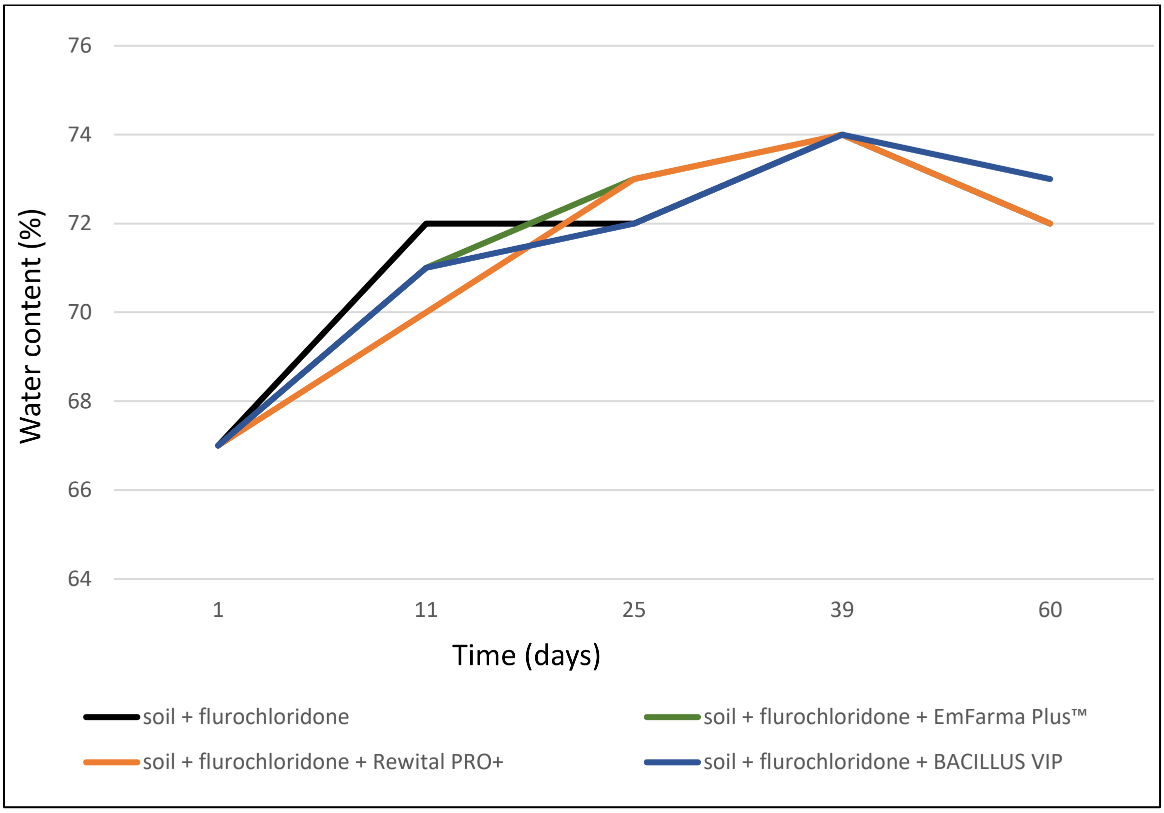

Figure S10: Changes in a moisture content of the soil with flurochloridone and following application of formulations with EM in time.

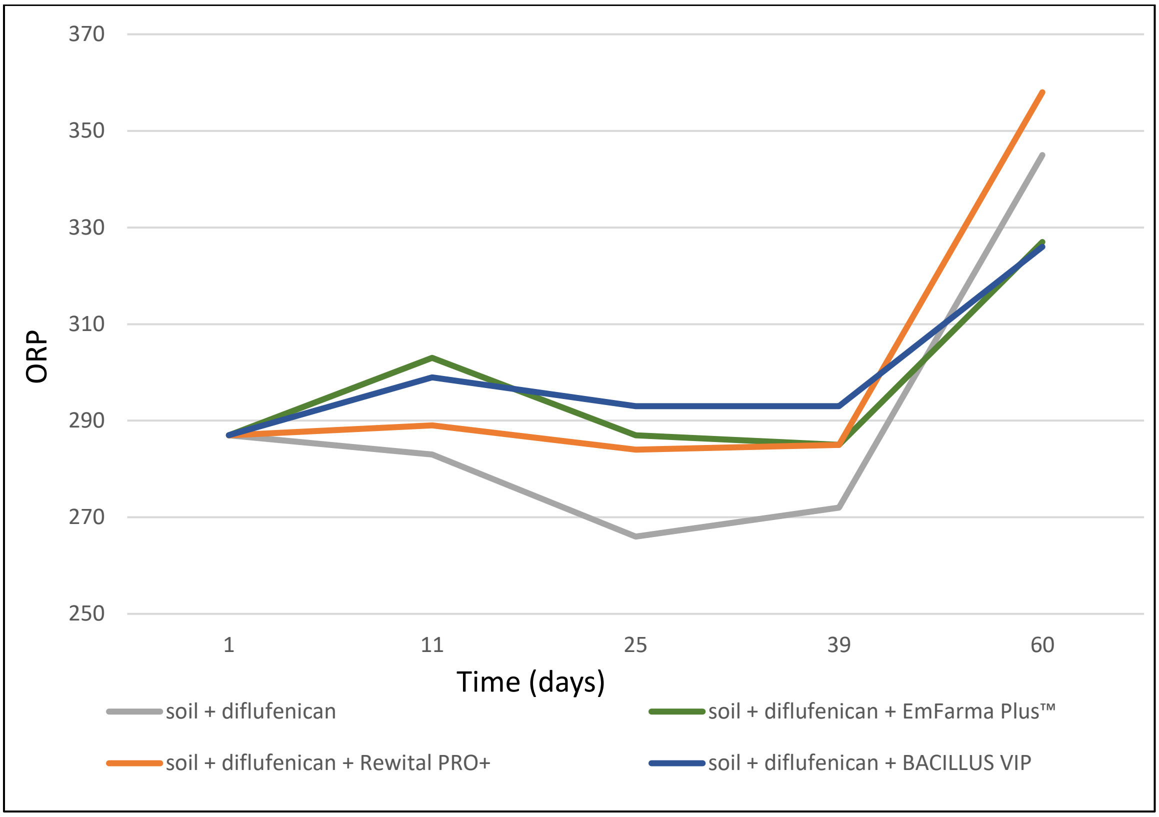

ORP — oxidation-reduction potential

Figure S11: Changes in the oxidoreduction potential of the soil with diflufenican and following application of formulations with EM in time.

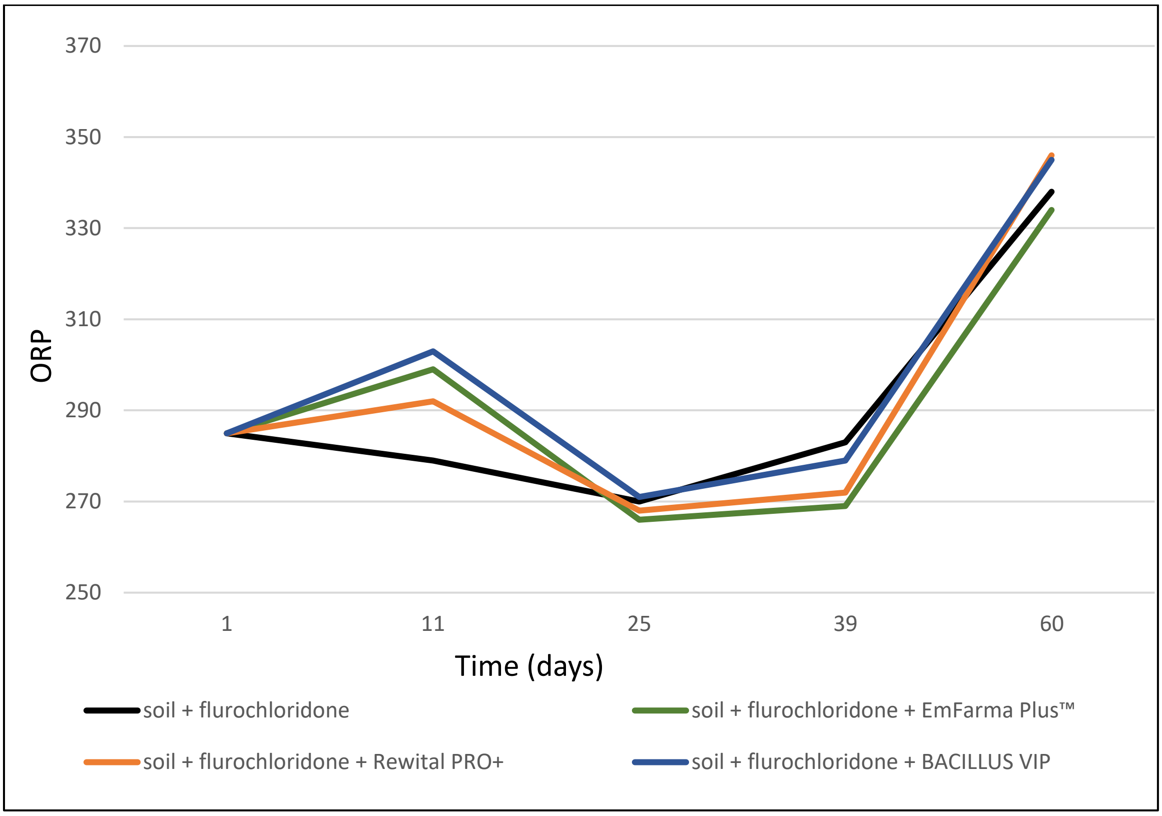

ORP — oxidation-reduction potential

Figure S12: Changes in the oxidoreduction potential of the soil with flurochloridone and following application of formulations with EM in time.

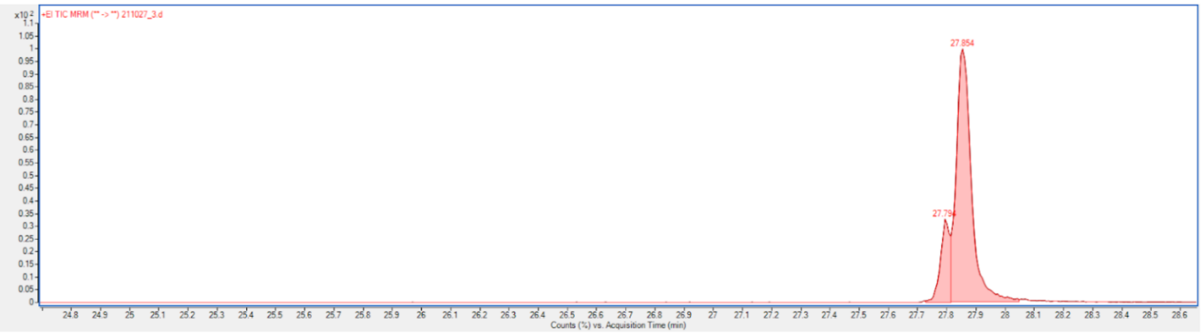

Figure S13: Chromatogram of soil sample with diflufenican (retention time for diflufenican – 27.854 min, and internal standard - triphenyl phosphate, TPP – 27.794 min).

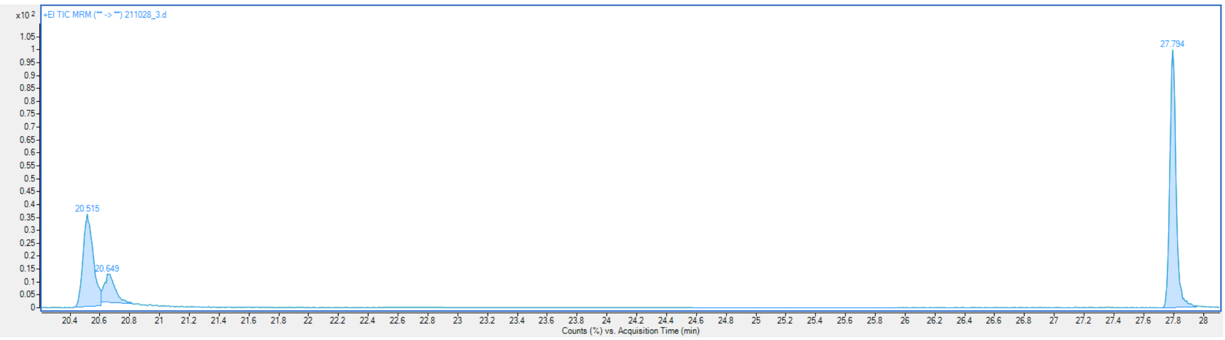

Figure S14: Chromatogram of soil sample with flurochloridone (retention time for flurochloridone – 20.515 min, and internal standard - triphenyl phosphate, TPP – 27.794 min).
